# Intraoperative application of low-dose dexmedetomidine or lidocaine for postoperative analgesia in pediatric patients following craniotomy: a randomized double-blind placebo-controlled trial

**DOI:** 10.3389/fsurg.2024.1371588

**Published:** 2024-06-24

**Authors:** Di Bao, Yaxin Wang, Wei Xiong, Di Zhang, Lanxin Qiao, Na Zheng, Lu Li, Xu Jin

**Affiliations:** ^1^ Department of Anesthesiology, Beijing Chaoyang Hospital, Capital Medical University, Beijing, China; ^2^ Department of Anesthesiology, Beijing Tiantan Hospital, Capital Medical University, Beijing, China; ^3^ Department of Anesthesiology, Cancer Hospital Chinese Academy of Medical Sciences, Beijing, China

**Keywords:** dexmedetomidine, lidocaine, pediatric, craniotomy, postoperative analgesia

## Abstract

**Background:**

Postoperative pain is a common occurrence in pediatric patients following craniotomy, often leading to negative outcomes. Intravenous dexmedetomidine and lidocaine are commonly used adjuvant medicines in general anesthesia to reduce perioperative opioid consumption and relieve postoperative pain in adults. While they show promise for use in pediatrics, the evidence of their application in pediatric craniotomy patients is limited. Therefore, we aimed to compare the effects of dexmedetomidine and lidocaine on postoperative pain in pediatric patients following craniotomy.

**Methods:**

We conducted a randomized, double-blind, single-center trial on children scheduled for craniotomy. The 255 recruited participants aged 1–12 years were randomly assigned to intraoperatively receive a loading intravenous dose of either dexmedetomidine 1 μg·kg^−1^ or lidocaine 2 mg·kg^−1^ or normal saline for 15 min followed by dexmedetomidine 0.5 μg·kg^−1^·h^−1^ or lidocaine 1 mg·kg^−1^·h^−1^ or normal saline until the sutures of endocranium were completed. The primary outcome was the cumulative sufentanil consumption within 24 h post-surgery.

**Results:**

A total of 241 patients were included in the statistical analysis. The primary outcome did not show any significant differences among the three groups (median (IQR) lidocaine group: 3.36 (1.32–5.64) μg vs. dexmedetomidine group: 3.12 (1.36–6.39) μg vs. control group 3.46 (1.77–7.62) μg, *p* = 0.485). Among the secondary outcomes, there was a statistically significant but small reduction in sufentanil consumption within 2 h, postoperative FLACC/WBFS/NRS pain scores within 4 h after surgery and postoperative Ramsay sedation scores in dexmedetomidine group (*p* < 0.05). Regarding postoperative complications, the incidence of electrolyte disturbance within 24 and 48 h after surgery was significantly higher in control group compared to the other two groups. There were no significant differences in intraoperative opioid consumption, postoperative frequency of remedy medication, or length of hospitalization among the three groups. No adverse events related to lidocaine or dexmedetomidine were observed.

**Conclusions:**

There were no significant differences in the primary outcome among the three groups. Although dexmedetomidine showed some benefits in reducing postoperative opioid consumption within the first 2 h and pain intensity within the first 4 h post-surgery, these findings should be interpreted with caution. Further research is required to comprehensively assess the outcomes and determine the optimal administration strategy.

**Clinical Trial Registration:**

[http://www.chictr.org.cn/index.aspx], identifier [ChiCTR1800019411].

## Background

Studies have reported that up to 60%–80% of adult patients experience moderate to severe pain within 24 h after craniotomy (1–3). Teo et al. reported that despite multimodal analgesia, 42% of pediatric patients still experienced at least one episode of moderate or severe pain within 72 h after craniotomy (4). Pain after craniotomy is associated with various side effects including agitation, intracranial hypertension, seizures and even postoperative hematoma, which further affects morbidity and mortality (5–8). However, postoperative pain in pediatric patients appears to be underestimated often due to limited pain expression and the difficulty of pain assessment. Additionally, the prevention and treatment of postoperative pain in pediatric craniotomy patients pose challenges due to unclear indications of medication in children and concerns about medication interfering with neurologic examinations. Therefore, proper perioperative analgesia management is crucial for pediatric neurosurgical patients. Currently, opioids are commonly used for postoperative analgesia, but they are associated with undesirable side effects such as increased risk of respiratory depression and postoperative nausea and vomiting, which can potentially elevate intracranial pressure and lead to intracranial hemorrhage in patients after neurosurgery (9). Multimodal analgesia, which combines opioids with other analgesic agents, is frequently employed to alleviate side effects. Intravenous lidocaine or dexmedetomidine are two promising agents in this regard.

Dexmedetomidine is a highly selective *α*_2_ adrenoreceptor agonist that can decrease perioperative opioid consumption and postoperative pain intensity in neurosurgery (10, 11). Additionally, it provides better control of intraoperative hemodynamic stability and has a unique neuroprotective effect (12–16). Lidocaine, a classical local anesthetic, is commonly used as a perioperative analgesic adjunct to enhance rehabilitation and promote better postoperative recovery in alduts (17–20). Similarly, studies have shown that intraoperative intravenous lidocaine can reduce opioid consumption and provide optimum analgesia in pediatric elective surgery (21–24). However, two recent meta-analyses have indicated that further research is needed to determine the effectiveness of lidocaine's analgesic effect (25, 26). Although both dexmedetomidine and lidocaine show promise for use in pediatrics, their analgesic effectiveness in pediatric patients following craniotomy has not yet been confirmed, necessitating further investigation. Therefore, this trial aims to explore and compare the postoperative analgesic effects of intravenous infusion of dexmedetomidine and lidocaine on pediatric patients scheduled for elective craniotomy. The results of this study will provide theoretical evidence for the use of multimodal analgesia in pediatric patients undergoing craniotomy.

## Method

### Study design

This study is a single-center, prospective, double-blinded, randomized controlled trial that was approved by the Institutional Review Board of the Beijing Tiantan Hospital, Capital Medical University on November 26, 2018 (No. KY 2018-087-02). It was registered at the Chinese Clinical Trial Registry (http://www.chictr.org.cn/index.aspx; No. ChiCTR1800019411) on November 28, 2018. The study was conducted from December 1, 2018 to December 14, 2021 at Beijing Tiantan Hospital, Capital Medical University.

The detailed protocol of this study has been published (27). Children aged 1–12 years old with ASA(American Society of Anesthesiologists) classification I–III, scheduled for elective craniotomy, were eligible for inclusion. Patients with a history of psychological disease, airway abnormalities, reactive airway diseases, other respiratory diseases, allergy to local anesthetics, sensitivity or contraindications to study medicines, liver or kidney dysfunction (defined as alanine aminotransferase or aspartate aminotransferase, blood urea nitrogen, or serum creatinine levels ≥1.5 times baseline), or combined with atrioventricular block diseases were excluded. Children who were unsuitable for extubation after the surgery were also excluded. All participants provided written informed consent. For children aged 1–6 years old, informed consent was obtained from their legal guardians. For children aged 7 years and over, written informed consent was obtained from both the child and the legal guardian.

### Randomization and blinding

Eligible children were randomly assigned to one of three groups: the dexmedetomidine group (group D), the lidocaine group (group L), or the control group (group N). The group assignments were determined using computer-generated random numbers prepared by a research assistant with no clinical involvement. The group allocation was concealed in an opaque sealed envelope with a serial number. After obtaining informed consent, an anesthetic nurse, who was not part of the trial, opened the envelope and prepared the study medications. Postoperative data in the PACU, ICU, or wards were collected by an independently trained anesthesiologist. The group assignments were blinded to the anesthesiologists, participants, and outcome assessors.

The study medicines contained either dexmedetomidine 4 μg·ml^−1^ or lidocaine 8 mg·ml^−1^ or normal saline in prefilled 50 ml syringes, and it was intravenously infused immediately after intubation by the blinded anesthesiologist. The infusion speed within the first 15 min was calculated according to the formula of 6*body weight/4 ml·h^−1^ followed by infusion speed of body weight/8 ml·h^−1^ until the sutures of the endocranium were completed. This rate corresponded to a loading intravenous dose of either dexmedetomidine 1 μg·kg^−1^ or lidocaine 2 mg·kg^−1^ or normal saline for 15 min followed by dexmedetomidine 0.5 µg·kg^−1^·h^−1^ or lidocaine 1 mg·kg^−1^·h^−1^. This approach resulted in three groups of patients receiving equal volumes of the study medicine per time.

### Safety

In this study, we only administered the study medicine using the safe dose that was reported in previous studies (28, 29) for safety considerations during the surgery. If any adverse events such as allergic reactions, systemic toxicity, or neurological dysfunction occurred during the infusion, the trial was immediately terminated and patient allocation was revealed. The anesthesiologists recorded and reported these events to the principal investigator. For patients who experienced harm as a result of their participation in the trial, free medical treatments and corresponding compensation were provided promptly.

### Anesthesia management

Standardized anesthesia management was provided to all children. Upon arrival in the operating room, standard monitoring was initiated and recorded. Children were premedicated with intravenous midazolam (0.025–0.075 mg·kg^−1^), and oral midazolam (0.5 mg·kg^−1^) was administered to those who were unable to allow peripheral venous access due to crying or irritability. Anesthesia was induced using sufentanil (0.3–0.5 μg·kg^−1^), propofol (1.5–2.5 mg·kg^−1^), and atracurium (0.15 mg·kg^−1^), followed by rapid sequence intubation. For children under 5 years of age with excessive anxiety, anesthesia was induced by 6%–8% sevoflurane inhalation before venous access. Intraoperatively, all patients received invasive arterial pressure monitoring.

Anesthesia was maintained with a totally intravenous infusion of propofol at a rate of 6–8 mg·kg^−1^·h^−1^ and remifentanil at a rate of 0.2–0.3 μg·kg^−1^·min^−1^, adjusted to maintain hemodynamics changes within 30% of the baseline values. Supply blood volume or use vasoactive medicine if necessary. Volume-controlled ventilation was performed with a tidal volume set at 8–10 ml kg^−1^ and the respiratory rate was adjusted to maintain the P_ET_CO_2_ between 35 and 45 mmHg. Approximately 30 min before the end of the surgery, sufentanil 0.1 µg·kg^−1^ was administered intravenously. Intraoperative armpit temperature was monitored and maintained at 35–37°C using thermal insulation measures. No additional analgesics were administered during the surgery, and there was no scalp nerve block or local infiltration anesthesia applied to the incision. All anesthetics were discontinued at the end of the surgery. After extubation, the children were transferred to the postoperative care unit (PACU).

An electronic analgesia pump (Apona® electronic infusion pump ZZB-I-150, APON Medical Technology Co., Ltd., Jiangsu, China) was used as a standard practice for postoperative pain management. The pump contained sufentanil 2 μg·kg^−1^ and ondansetron 0.3 mg·kg^−1^, which were diluted in 100 ml of normal saline. It delivered a bolus of 2 ml with a 30 min lock-out time and did not have a continuous background infusion. In the post-anesthesia care unit (PACU), intensive care unit (ICU), or general ward, children between the ages of 1 and 6 years received nurse-controlled analgesia when their FLACC score was ≥4. For children aged 7–12 years, nurses assisted them in pain relief until they were able to operate the device independently. If the FLACC pain score was >5 or the FACES pain score was ≥6, intravenous acetaminophen was administered as a remedial measure, with an initial dose of 15 mg·kg^−1^. The parameters of the electronic analgesia pump, such as the total dosage of sufentanil and the number of compressions, as well as the initiation of emergency rescue measures (including agent dosage and frequency), were recorded within 48 h after surgery.

### Outcome measurement and data collection

The primary outcome of this study was the cumulative sufentanil consumption administered via the electronic analgesia pump within the first 24 h after the surgery. The secondary outcomes included: (1) the consumption of sufentanil and the frequency of remedy medication within 1 h, 2 h, 4 h, and 48 h after the surgery; (2) postoperative pain scores at 1 h, 2 h, 4 h, 24 h, and 48 h.To comprehensively assess postoperative pain in children, we utilized three different pain assessment scales: the Face, Leg, Activity, Crying and Consolability (FLACC) scale (26), Wong-Baker Faces Pain-rating Scale (FACES) (30), and the numerical rating scale (NRS). The FLACC and FACES scales are suitable for children aged 1–12 years, while the NRS scale is appropriate for children aged 7–12 years. (3) the requirement for opioids during anesthesia; (4) the incidence of postoperative complications, including intracranial infection, neurosurgery-related complications, postoperative nausea and vomiting (PONV), and severe adverse reactions (SAE) such as disability and death within 24 h and 48 h after the surgery; (5) Ramsay Sedation Score. We evaluated the postoperative sedation status of the patients considering the sedative effects of dexmedetomidine. The Ramsay sedation score, which has been demonstrated to effectively reflect postoperative sedation in children (22), was used for this evaluation. (6) the length of hospitalization, recorded as the number of nights spent in the hospital after the surgery.

The baseline data included: (1) demographic data such as gender, age, height, and weight, which were collected directly through the anesthesia information management system; (2) ASA classification; and (3) surgical characteristics, such as craniotomy approach, lesion site, tumor characteristics, and whether a ventriculoperitoneal shunt was performed before craniotomy.

The perioperative data includes vital signs such as heart rate (HR) and mean artery pressure (MAP), which were collected at six different time points: upon entering the operating room, during intubation, during skin incision, at the end of the suture, 5 min before extubation, and 5 min after extubation. Other data collected include anesthetic management data, such as the total consumption of anesthetics like propofol, remifentanil, and sufentanil. Additionally, several important time points were recorded, including the start and end time of the surgery, the patient’s awake time, and the extubation time. Furthermore, information on fluids administered, intraoperative blood loss, and urine output was also documented.

### Sample size and statistical analysis

We calculated the planned sample size was 255 patients as stated in the protocol (27). All data were processed using SPSS24.0 and summarized using appropriate descriptive statistics. Variables are presented as numbers (percentages), means (SD) or medians [interquartile range (IQR)]. The Kolmogorov–Smirnov tests were performed to detect the normal distribution of continuous variables. Continuous data were analyzed using the Student's *t*-test, ANOVA (One-Way Analysis of Variance) or repeated measures ANOVA. Categorical data were analyzed using the chi-square test or Fisher's exact test. For the primary outcome, non-parametric tests were used to compare statistical differences between the three groups. In cases where statistical differences were found, multiple comparisons were further conducted. Subgroup analysis was performed based on different age groups. Subjects who refused intervention or had missing data related to the primary outcome were excluded from the final statistical analysis. A *p*-value of less than 0.05 was considered statistically significant among the three groups.

## Results

**Figure 1:**
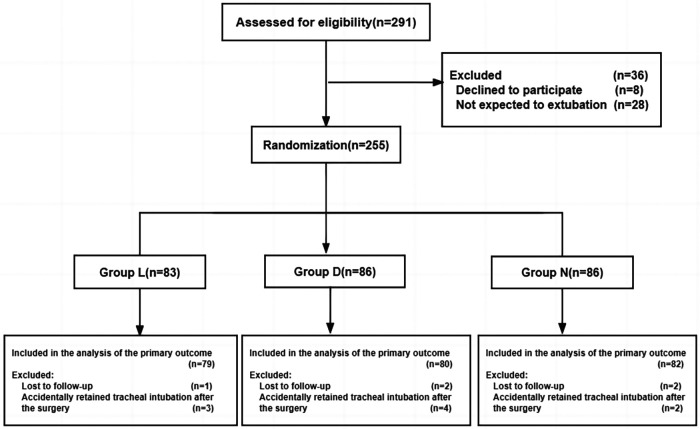
Flow chart of paticipants. Group L: the lidocaine group; Group D: the dexmedetomidine group; Group N: the control group.

From December 1, 2018 to December 14, 2021, a total of 291 pediatric patients were screened for eligibility (as shown in Figure 1). Among them, 36 patients were excluded, including 8 patients or their legal guardians who refused to participate, and 28 patients who were not expected to undergo extubation after surgery. Eventually, 255 pediatric patients proceeded to randomization. A total of 17 patients were later excluded, including 9 patients who accidentally retained tracheal intubation after surgery, and 5 patients who lost follow-up for the primary outcome. Finally, 241 subjects were included in the statistical analysis, with 79 patients receiving lidocaine, 80 patients receiving dexmedetomidine, and 82 patients receiving placebo. The flow chart of the study is presented in Figure 1. Baseline characteristics among the three groups were comparable and are detailed in Table 1.

**Table 1:** Baseline demographic and clinical characteristics.

Characteristic	Group L (*n* = 79)	Group D (*n* = 80)	Group N (*n* = 82)	*P* value
Sex (male,%)	46 (58.2)	43 (53.8)	44 (53.7)	0.803
Age (year)	6.44 ± 2.99	5.83 ± 3.37	6.17 ± 3.37	0.487
Age [*n* (%)]				
1–6 years old	40 (16.5)	41 (17.0)	41 (17.0)	0.354
7–12 years old	39 (16.2)	39 (16.2)	41 (17.0)	
Weight (kg)	23 (18–28)	22.75 (15–34.5)	21.5 (16–35)	0.909
BMI (kg/m^2^)	16.09 (14.70–18.28)	16.54 (15.36–18.49)	16.16 (14.71–19.68)	0.548
HR on admission (beats/min)	86.20 ± 9.70	86.76 ± 11.16	84.78 ± 12.07	0.500
MAP on admission (mmHg)	78.02 ± 6.68	78.57 ± 8.20	77.01 ± 8.08	0.42
ASA				
I [*n* (%)]	0 (0)	0 (0)	0 (0)	/
II [*n* (%)]	79 (100)	80 (100)	82 (100)	
III [*n* (%)]	0 (0)	0 (0)	0 (0)	
Craniotomy approach [*n* (%)]				
Transfrontal approach	31 (39.2)	24 (30.0)	39 (48.1)	0.442
Transtemporal approach	13 (16.5)	16 (20.0)	13 (16.0)	
Parietal occipital approach	6 (7.6)	7 (8.8)	7 (8.6)	
Posterior median approach	27 (34.2)	32 (40.0)	22 (27.2)	
Other	2 (2.5)	1 (1.2)	0 (0)	
Shunt surgery (%)	17 (21.8)	13 (16.2)	19 (23.2)	0.515
Tumor site [*n* (%)]				
Frontal part	8 (10.1)	7 (8.8)	7 (8.5)	0.707
Tempus	6 (7.6)	6 (7.5)	7 (8.5)	
Occipital-parietal	6 (7.6)	6 (7.5)	4 (4.9)	
Sellar	15 (19.0)	10 (12.5)	22 (26.8)	
Cerebellum	17 (21.5)	20 (25.0)	14 (17.1)	
Ventricles	19 (24.1)	26 (32.5)	24 (29.3)	
Other	8 (10.1)	5 (6.2)	4 (4.9)	
Classification of tumor site [*n* (%)]				
Supratentorial	38 (48.1)	33 (41.2)	46 (56.1)	0.167
Infratentorial	41 (51.9)	47 (58.8)	36 (43.9)	
Tumor classification [*n* (%)]				
Glioma	42 (53.2)	39 (48.8)	32 (39.0)	0.078
Medulloblastoma	11 (13.9)	19 (23.8)	10 (12.2)	
Craniopharyngioma	13 (16.5)	9 (11.2)	19 (23.2)	
Other	13 (16.5)	13 (16.2)	21(25.6)	
Abnormal hormone level [*n* (%)]	12(16.4)	5(6.2)	15(18.3)	0.065

Data are expressed as mean standard deviation, median (IQR), or number of cases (percentage).

### Intraoperative characteristics

As presented in Supplementary Table S1, patients’ intraoperative characteristics were comparable except the urine output in group D was significantly higher compared to the other two groups (median (IQR) group D: 600 (500–1,100) ml vs. group L: 800 (525–1,200) ml vs. group N: 575 (350–1,000) ml; *p* = 0.003).

Hemodynamic changes at different time points among the three groups are shown in Supplementary Figures S1,S2. Intraoperative hemodynamics were relatively stable in all three groups. From the time of skin incision to 5 min after extubation, the heart rate in group D was lower than in the other groups, and it was significantly lower at the end of the suture and 5 min before and after extubation (*p* < 0.05).

### Primary outcome

**Figure 2:**
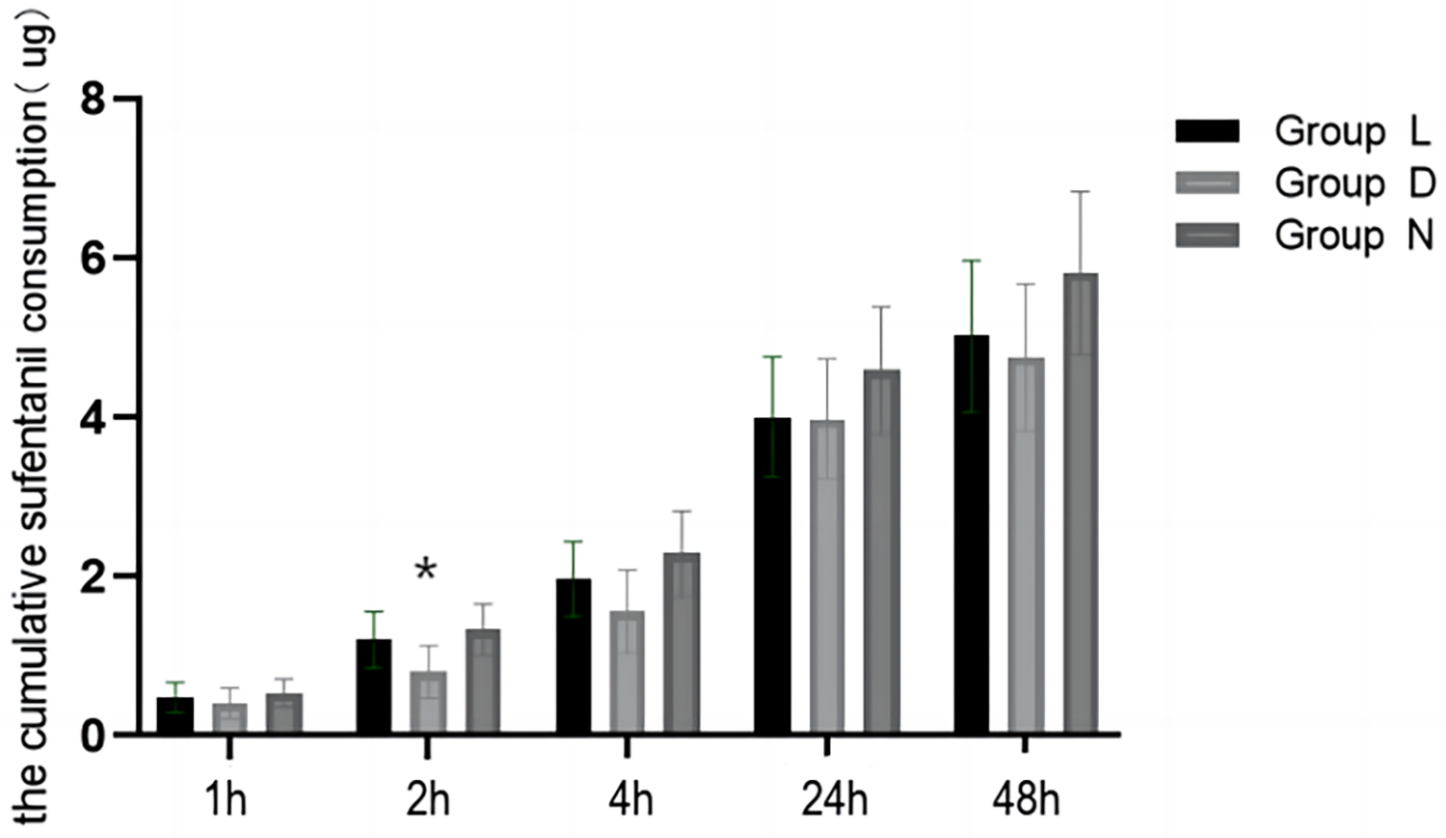
Comparison of sufentanil consumption in patients aged 1–12 years old at different time points. Group L: the lidocaine group; Group D: the dexmedetomidine group; Group N: the control group.(*p < 0.05, comparison between group D and group N).

A total of 241 patients were included in the statistical analysis of sufentanil consumption within 24 h after surgery (Figure 2; Table 2). The results indicated that the consumption of sufentanil within this timeframe was lower in group D and group L compared to group N. However, there was no statistically significant difference observed among the three groups (median (IQR) group L: 3.36 (1.32–5.64) μg vs. group D: 3.12 (1.36–6.39) μg vs. group N: 3.46 (1.77–7.62) μg, *p* = 0.485). Subgroup analysis was conducted based on different age groups (1–6 years old and 7–12 years old). Supplementary Table S2 demonstrates that there was no statistically significant difference observed after Bonferroni-corrected *post hoc* pairwise comparisons. Furthermore, the non-parametric test revealed a statistically significant difference in sufentanil consumption 24 h after surgery among different surgical craniotomy methods (*p* < 0.05). Specifically, the consumption of sufentanil during posterior median craniotomy was significantly higher than that during transfrontal craniotomy (*p* < 0.05).

**Table 2:** Primary outcome and secondary outcomes.

	Group L (*n* = 79)	Group D (*n* = 80)	Group N (*n* = 82)	*P* value
Primary outcome				
Sufentanil consumption in 24 h after surgery	1.74 (0.6–2.82)	1.37 (0.42–3.90)	1.64 (0–4.48)	0.879
Secondary outcomes (μg) from end of surgery to exit from the room				
Respiratory recovery time (min)	10 (7–15.5)	11 (6.75–19.50)	10.50 (6–17)	0.658
Awake time (min)	31 (19–47.50)	48 (28.75–74)	33 (24–48)	*0.000
Time to extubation (min)	21 (14–32.50)	26 (17–36.50)	21 (14–29)	0.127
Complications in recovery period [*n* (%)]				
agitation	6 (7.6)	5 (6.2)	7 (8.5)	0.684
nausea	6 (7.6)	1 (1.2)	2 (2.4)	0.081
vomit	4 (5.1)	2 (2.5)	1 (1.2)	0.337
1 h after surgery				
Ramsay sedation score	3 (2–3)	3 (2–4)	3 (2–4)	*0.000
Remedial agents [*n* (%)]	2 (2.5)	2 (2.5)	2 (2.4)	0.999
Postoperative complications [*n* (%)]				
PONV	9 (11.4)	7 (8.8)	7 (8.5)	0.792
Neurological complications	5 (6.3)	15 (18.8)	15 (18.3)	0.078
Intracranial infection	0 (0)	0 (0)	0 (0)	/
Electrolyte disorder	2 (2.5)	1 (1.3)	0 (0)	0.346
Emergency CT	0 (0)	0 (0)	0 (0)	/
2 h after surgery				
Remedial agents [*n* (%)]	0 (0)	0 (0)	0 (0)	/
Postoperative complications [*n* (%)]				
PONV	11 (13.9)	9 (11.2)	8 (9.8)	0.626
Neurological complications	5 (6.3)	10 (12.5)	12 (14.6)	0.224
Intracranial infection	0 (0)	0 (0)	0 (0)	/
Electrolyte disorder	1 (1.3)	1 (1.3)	1 (1.2)	0.918
Emergency CT	0 (0)	0 (0)	0 (0)	/
4 h after surgery				
Remedial agents [*n* (%)]	1 (1.3)	2 (2.5)	0 (0)	0.357
Postoperative complications [*n* (%)]				
PONV	8 (10.1)	12 (15.0)	6 (7.3)	0.281
Neurological complications	3 (3.8)	7 (8.8)	3 (3.7)	0.267
Intracranial infection	2 (2.5)	0 (0)	0 (0)	0.126
Electrolyte disorder	6 (7.6)	11 (15.0)	17 (20.7)	0.060
Emergency CT	0 (0)	0 (0)	0 (0)	/
24 h after surgery				
Remedial agents [*n* (%)]	1 (1.3)	0 (0)	1 (1.2)	0.606
Postoperative complications [*n* (%)]				
PONV	11 (13.9)	5 (6.2)	10 (12.2)	0.323
Neurological complications	1 (1.3)	6 (7.5)	4 (4.27)	0.207
Infect	7 (8.9)	9 (11.5)	3 (3.7)	0.186
Electrolyte disorder	16 (20.3)	11 (13.8)	40 (48.8)	*0.000
Emergency CT	1 (1.3)	2 (2.5)	0 (0)	0.357
48 h after surgery				
Remedial agents [*n* (%)]	0 (0)	1 (1.2)	2 (2.4)	0.378
Postoperative complications [*n* (%)]				
PONV	8 (10.1)	3 (3.8)	2 (2.4)	0.059
Neurological complications	2 (2.5)	4 (5.1)	1 (1.2)	0.348
Intracranial infection	7 (8.9)	11 (15.0)	8 (10.1)	0.386
Electrolyte disorder	11 (13.9)	17 (21.2)	28 (34.1)	*0.009
Emergency CT	2 (2.5)	2 (2.5)	0 (0)	0.350
Length of postoperative hospitalization (days)	13 (10–18)	12 (10–14)	13 (9–16)	0.609

Data are expressed as median (IQR) or the number of cases (percentage).

*p < 0.05; Diagnostic criteria for intracranial infection: Diagnosis requires meeting at least 5 criteria: (1) Presence of clinical symptoms and signs of intracranial infection, such as body temperature >38°C, headache, consciousness disorders, etc. (2) Elevated infection indicators, including blood white blood cell count >9.5*10^9 ^/L or neutrophil ratio >80%, CRP (C-reactive protein) >8.2 mg/L or PCT (procalcitonin) >0.25 ng/ml, etc. (3) Cerebrospinal fluid examination revealing white blood cell levels >10*10^7^ /L or protein level >0.45 g/L. (4) CT or MRI scans of the head showing diffuse edema, subdural abscess, or subdural abscess in the brain. (5) Turbidity of cerebrospinal fluid during lumbar puncture. (6) Identification of pathogenic bacteria on cerebrospinal fluid smear. (7) Positive bacterial culture in cerebrospinal fluid.Neurological complications may include the occurrence of postoperative epilepsy and postoperative diabetes insipidus.

### Secondary outcomes

#### Sufentanil consumption

The consumption of sufentanil in group D after the surgery was lower compared to the other groups. However, this difference was statistically significant only at 2 h and 4 h (median (IQR) group L: 0.68 (0–2.0) μg vs. group D: 0 (0–1.10) μg vs. group N: 0.74 (0–2.62) μg, *p* = 0.005) (median (IQR) group L: 1.36 (0.46–3.08) μg vs. group D: 0.76 (0–2.06) μg vs. group N: 1.54 (0–3.88) μg, *p* = 0.043). After performing bonferroni-corrected *post hoc* pairwise comparisons, it was found that the sufentanil consumption within 2 h postoperatively was significantly lower in group D compared to group D, but there was no significant difference within 4 h after the surgery. The frequency of remedy medication after postoperative compression analgesia pumps was low (4.1%), and there was no significant difference among the three groups at any time point.

#### Postoperative pain scores

Supplementary Figures S3,S4 present a comparison of FLACC and WBFS pain scores among patients aged 1–12 years at different time points. The pain scores gradually decrease from 4 h to 48 h after surgery. Group D exhibited significantly lower pain scores of FLACC/WBFS compared to the other two groups after extubation, 1 h, and 2 h post-surgery, as indicated by the Kruskal–Wallis test (*p* < 0.05) (Supplementary Figure 5).

#### Opioid consumption during anesthesia

To facilitate calculation, the amount of sufentanil used during the surgery was converted to the equivalent amount of remifentanil using standardized conversion factors, with a ratio of 1:10. The Kruskal–Wallis H test revealed no significant difference in the intraoperative consumption of opioids among the three groups. The median (IQR) consumption in the L group was 177.5 (133.60–265.00) μg, in the D group was 153.10 (117.50–237.00) μg, and in the N group was 170.50 (137.75–275.70) μg (*p* = 0.238).

#### The ramsay sedation score

There was a statistically significant difference in Ramsay sedation score among the three groups 1 h after surgery (median (IQR) group D: 3 (2–3) vs. group L: 3 (2–4) vs. group N: 3 (2–4); *p* = 0.000). *post hoc* pairwise comparison revealed that group D had significantly lower scores compared to the other groups (*p* = 0.001, *p* = 0.002). The three groups also showed significant differences in awake time (median (IQR) group D: 31 (19–47.50) min vs. group L: 48 (28.75–74) min vs. group N: 33 (24–48) min; *p* = 0.000). Further analysis indicated that group D had significantly longer awake hours compared to the other two groups, and this difference was statistically significant (*p* = 0.005).

#### Postoperative complications and length of hospitalization

There were no statistically significant differences in the incidence rates of neurological-related complications, the need for emergency CT, and intracranial infection among the three groups. However, there was a statistical difference in the incidence of electrolyte imbalance between the three groups at 24 h and 48 h after surgery. Specifically, group N had a significantly higher incidence compared to group D or group L (24 h after surgery: group N *p* = 0.000, *p* = 0.000; 48 h after surgery: *p* = 0.048, *p* = 0.002). No allergic reactions, systemic toxicity, or neurologic dysfunction related to lidocaine or dexmedetomidine were reported during anesthesia or within 48 h of endotracheal intubation removal.

## Discussion

For the first time in our study, we investigated and compared the impact of intraoperative infusion of low-dose dexmedetomidine or lidocaine on postoperative pain in children undergoing craniotomy. Our primary outcome was the consumption of sufentanil within 24 h after surgery, and we found no statistically significant difference among the three groups. There are several possible reasons for this outcome. Firstly, the low incidence of moderate to severe pain after craniotomy in our trial resulted in less postoperative sufentanil consumption, which may explain the lack of significant difference in the primary outcome. Secondly, due to their limited ability to express pain, the pain after surgery is frequently inappropriately considered associated with emotional responses such as lack of parental companionship, retention of urinary catheters after surgery, etc (31–33), leading to lower sufentanil consumption. Additionally, the short elimination half-life of dexmedetomidine (approximately 2 h) suggests a limited duration of action, which could have contributed to the negative results in our primary outcome.

While previous studies have demonstrated the analgesic and anti-inflammatory benefits of intravenous lidocaine in various surgeries (28, 34–37), its analgesic effectiveness during surgery remains inconclusive and inconsistent across different surgical procedures (38). A meta-analysis revealed significant pain relief in patients undergoing abdominal surgery, but not in those undergoing other types of surgeries (25). In 2018, Weibel S et al. conducted a meta-analysis of 68 studies involving over 4,500 patients to investigate the effect of perioperative intravenous lidocaine infusion compared with placebo or epidural analgesia on postoperative pain and recovery in adults, and concluded with low-quality evidence that intraoperative infusion of lidocaine can reduce pain intensity 1–4 h after surgery, which is equivalent to an average pain reduction of 0.37 cm–2.48 cm on a 0–10 cm visual analog scale. It concluded that the analgesic effect of lidocaine after surgery remains uncertain, and no significant subgroup differences between different types of surgery in this study (26). In our study, we found no statistically significant differences between group L and group N in FLACC/WBFS/NRS postoperative pain scores, intraoperative and postoperative opioid consumption, or postoperative complication rates, which failed to prove the effectiveness of intraoperative infusion for analgesia in children undergoing craniotomy.

Two meta-analyses have shown that dexmedetomidine enhanced the analgesic effect of opioids, reducing perioperative and PACU opioid consumption and postoperative pain intensity (10, 39). Wang L et al. conducted a recent meta-analysis including 22 trials on the analgesic effect of dexmedetomidine as an adjunct to general anesthesia in adult craniotomy. The results indicated that compared with placebo, low-quality evidence showed dexmedetomidine did not significantly reduce postoperative opioid consumption; high to moderate quality evidence suggests that although dexmedetomidine produces a small statistically significant reduction in postoperative PACU pain intensity, it is not clinically significant (29). In our study, we observed that the dexmedetomidine group had lower postoperative consumption of sufentanil compared to the other groups, which was statistically significant at 2 h after surgery. Although the pain scores within 1–4 h after surgery were significantly lower in the dexmedetomidine group compared to the other two groups, the median difference in all pain scale scores was less than 3 points, indicating a small clinical significance. Therefore, our findings suggest that dexmedetomidine has limited analgesic effect in pediatric patients undergoing craniotomy. Compared to the other two groups, the dexmedetomidine group had a significantly longer postoperative awake time and a significantly higher Ramsay sedation score 1 h after surgery. It is believed that the analgesic and sedative effects of dexmedetomidine may be partially attributed to reduced anxiety and altered perception. The specific mechanism behind the analgesic effects is still unclear and complex, but current understanding suggests that it is mediated by a_2_-receptor binding in the brain and spinal cord (40). Additionally, research has demonstrated that dexmedetomidine has a significant anti-inflammatory effect, which can attenuate the perioperative secretion of inflammatory cytokines (IL, TNF-α) and inhibit postoperative inflammatory responses (14–16). Moreover, dexmedetomidine appears to enhance the analgesic effect of opioids through synergistic action (39, 41, 42).

In this study, most children experienced mild to moderate pain after craniotomy and the incidence of severe pain was 9.5%(any rating scale score ≥7). The frequency of remedial medication after applying the analgesic pumps was very low (4.1%), which supports the effectiveness of analgesia in our trial. Postoperative pain scores change with time in three groups, with pain intensity decreasing 24–48 h after surgery. Our study indicates that the craniotomy site was related to the consumption of sufentanil after surgery, of which the consumption of sufentanil was the largest after the transtemporal craniotomy and posterior midline craniotomy. The difference in sufentanil consumption between the posterior midline craniotomy and transfrontal craniotomy was statistically significant. The reason may be that more local muscle damage and soft tissue stretching in the posterior midline approach (43) leads to an increase in the incidence of postoperative pain and the consumption of sufentanil after the surgery. Therefore, for pediatric neurosurgery patients undergoing posterior midline craniotomy, multimodal analgesia should be combined in the perioperative period to effectively prevent postoperative analgesia.

Previous literature has reported that bradycardia is the most common adverse event during dexmedetomidine infusion (38, 42). To reduce the incidence of bradycardia, we opted for a lower-loading dose and infused it for 15 min. In our study, we found that the intraoperative heart rate in the dexmedetomidine group was statistically lower than in the control group. However, it is important to note that the observed bradycardia did not have any negative impact on perfusion or necessitate significant clinical intervention. Since our study only included healthy children, we recommend exercising caution when using dexmedetomidine in children with rate-dependent cardiac output. Additionally, we observed a significantly greater intraoperative urine output in the dexmedetomidine group compared to the control group. This can be attributed to the activation of the *α*_2_ receptor, which centrally inhibits the secretion of antidiuretic hormone (44). Furthermore, dexmedetomidine's peripheral inhibition of renin secretion increases the glomerular filtration rate and the secretion of water and sodium (45), ultimately leading to increased urine output.

The incidence of postoperative nausea and vomiting (PONV) in children is reported to be approximately 30%–80% (46). This study observed a low incidence of nausea and vomiting within 24 h after surgery in all three groups, with an overall incidence of 9.5%. It is possible that this incidence is influenced by the type of surgery. There were no significant statistical differences between the groups in terms of postoperative complications such as PONV, postoperative neurological complications, intracranial infection, emergency CT(Computed Tomography), and length of hospitalization. However, within 24 h and 48 h after surgery, group N had a significantly higher incidence of electrolyte disorders compared to the other groups. This could be attributed to the higher proportion of patients with craniopharyngioma in group N. Craniopharyngioma lesions are typically located in the sellar region and often involve the pituitary or hypothalamus, leading to a higher risk of electrolyte imbalance due to temporary or permanent reduction in hypothalamic and pituitary function after surgery (47, 48). No occurrences of allergic reactions, systemic toxicity, or neurological dysfunction related to lidocaine or dexmedetomidine were reported during anesthesia or within 48 h of tracheal intubation removal, indicating their safety for intraoperative infusion.

This study has several limitations. Firstly, we only administered experimental agents preoperatively and intraoperatively at safe doses based on previous studies (29, 49–51). We did not evaluate the effects of different doses of lidocaine or dexmedetomidine on postoperative pain. Secondly, due to technical limitations, we were unable to evaluate the effect of intravenous lidocaine on the neuroendocrine response to surgical trauma using objective biochemical markers such as blood levels of cytokines and catecholamines. Thirdly, the long duration of neurosurgery and limited postoperative follow-up time resulted in the assessment of sufentanil consumption and postoperative pain scores at 1 h, 2 h, 4 h, 24 h, and 48 h after surgery with large intervals. This can be further refined for more accurate results. In addition, we conducted at a single-centered and thus we cannot exclude the possibility of single-centered effects. Finally, while children aged 7–12 years have the capability to self-evaluate using NRS and utilize analgesia pumps, it is important to note that some children were apathetic after neurosurgery. Consequently, the dependability of NRS self-assessment and the evaluation of analgesic agent consumption after neurosurgery in children may be compromised. In the future, additional research centers and randomized controlled studies with larger sample sizes may be needed to further explore the benefits of dexmedetomidine on postoperative pain scores, opioid consumption, and postoperative complications.

## Conclusion

This trial is the first study to investigate the effect of intraoperative administration of low-dose dexmedetomidine or lidocaine on postoperative pain in children undergoing craniotomy. While there was no significant difference in postoperative cumulative sufentanil consumption within 24 h after craniotomy among the three groups, dexmedetomidine showed slight benefits in reducing postoperative opioid consumption and pain intensity compared to the control group. In contrast, intraoperative infusion of lidocaine did not demonstrate a significant reduction in opioid consumption, pain intensity, or postoperative complications. These findings suggest that dexmedetomidine may be a valuable component of a multimodal analgesic strategy for enhancing recovery in pediatric craniotomy patients. However, further evidence is needed to comprehensively evaluate the related clinical outcomes and determine the optimal administration strategy.

## Data Availability

The raw data supporting the conclusions of this article will be made available by the authors, without undue reservation.
