# Supplementary material for: Intraoperative application of low-dose dexmedetomidine or lidocaine for postoperative analgesia in pediatric patients following craniotomy: a randomized double-blind placebo-controlled trial
Source: Front Surg. 2024 Jun 24;11:1371588. doi: 10.3389/fsurg.2024.1371588 (PMC11228272; doi:10.3389/fsurg.2024.1371588)
Supplement: Supplementary file 1 [file Datasheet1.docx]

Supplementary Table 1 Surgical and intraoperative characteristics.

|  | **Group L (n=79)** | **Group D (n=80)** | **Group N**  **(n=82)** | **P value** |
| --- | --- | --- | --- | --- |
| Duration of surgery (min) | 270[227-314] | 251[216-316] | 250[187.75-302.50] | 0.241 |
| Duration of anesthesia (min) | 365[305-432] | 334[286.75-425.50] | 352[270-410.25] | 0.399 |
| Crystal solution (ml) | 1000[800-1300] | 1000[750-1400] | 925[600-1262] | 0.054 |
| Colloidal fluid (ml) | 200[100-250] | 150[100-300] | 200[0-300] | 0.732 |
| Intraoperative blood transfusion (n [%]) | 25(27.3） | 32(40) | 26(31.7) | 0.440 |
| Plasma (n [%]) | 15(19.0) | 19(23.8) | 14(17.1) | 0.550 |
| Intraoperative blood loss (ml) | 100[100-200] | 100[100-200] | 100[100-200] | 0.883 |
| Urine volume (ml) | 600[500-1100] | 800[525-1200] | 575[350-1000] | *0.003 |
| Intraoperative propofol consumption (mg) | 900[662-1192.5] | 795[537.5-1201.25] | 767.5[565-1242.5] | 0.487 |
| Intraoperative remifentanil consumption (μg) | 1724[1200-2468] | 1410[1078.40-2090] | 1485[1142-2575] | 0.167 |
| Total intraoperative sufentanil consumption (μg) | 15[12-22.75] | 15[11.38-24.25] | 16[10.0-23] | 0.949 |
| Intraoperative opioid consumption (converted into sufentanil consumption) (ug) | 177.5[133.60-265.00] | 153.10[117.50-237.00] | 170.50[137.75-275.70] | 0.238 |

Data are expressed in median [IQR] or number of cases (percentage); *p<0.05

Supplementary Table 2 Analysis results of primary outcome of different age groups

|  | **Group L (n=79)** | **Group D (n=80)** | **Group N(n=82)** | **P value** |
| --- | --- | --- | --- | --- |
| 1-6 years old (n=122) | 2.40[0.84-4.54] | 2.00[1.16-3.60] | 2.00[0.64-3.20] | 0.444 |
| 7-12 years old (n=119) | 4.96[1.44-8.00] | 5.92[2.24-7.84] | 7.60[4.34-9.46] | 0.044 |


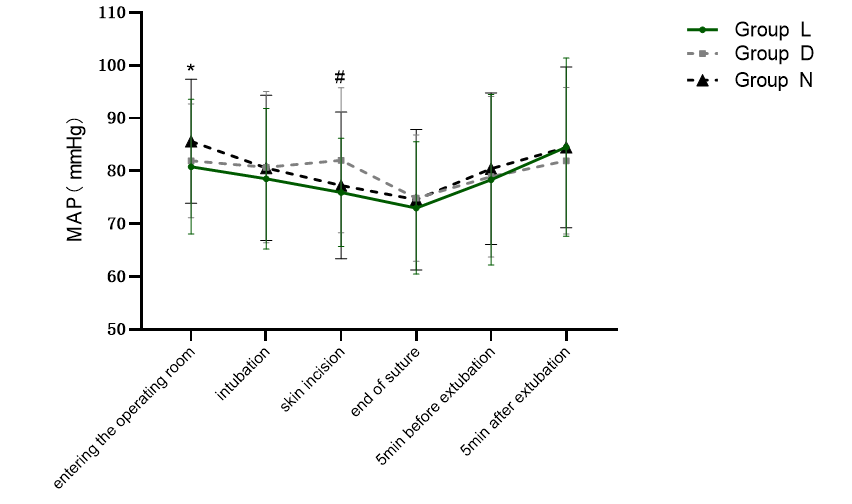


Supplementary Figure. 1 Comparison of MAP of patients aged 1-12 years at different time points (*p<0.05,comparison between group L and group N; #p<0.05, comparison between group L and group D)


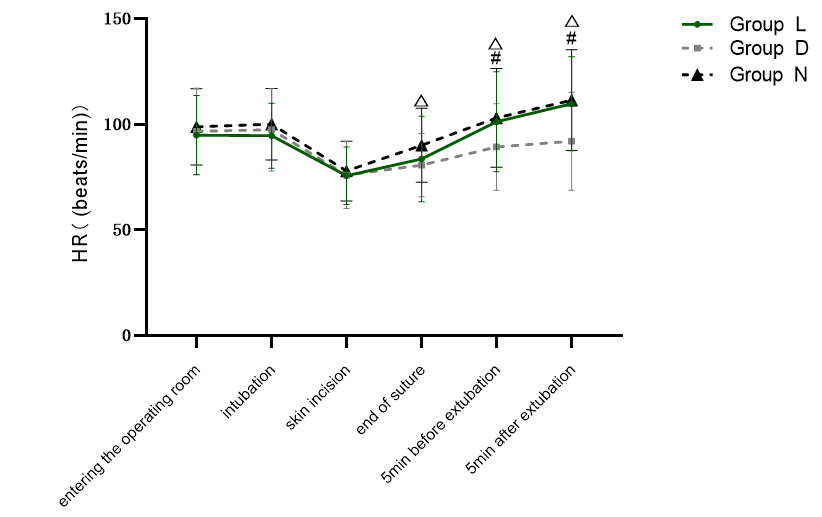


Supplementary Figure. 2 Comparison of heart rate of patients aged 1-12 at different time points.

(△p<0.05, comparison between group D and group N; #p<0.05 comparison between group L and group D)


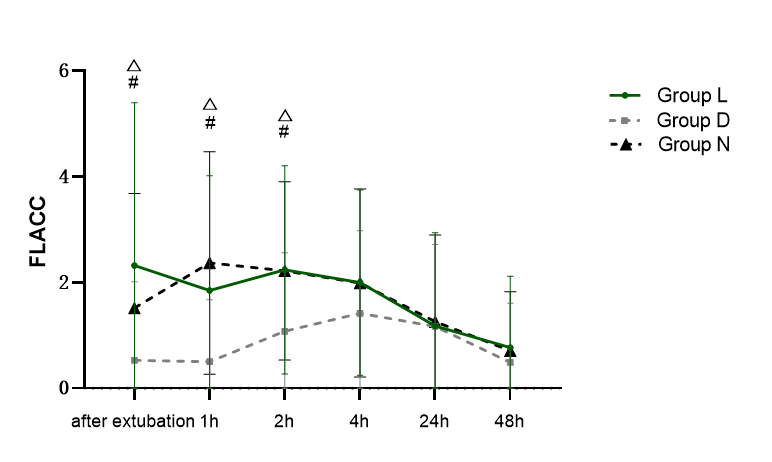


Supplementary Figures 3 Comparison of FLACC pain scores of patients aged 1-12 years old at different time points (△p<0.05, comparison between group D and group N; #p comparison between group L and group D)


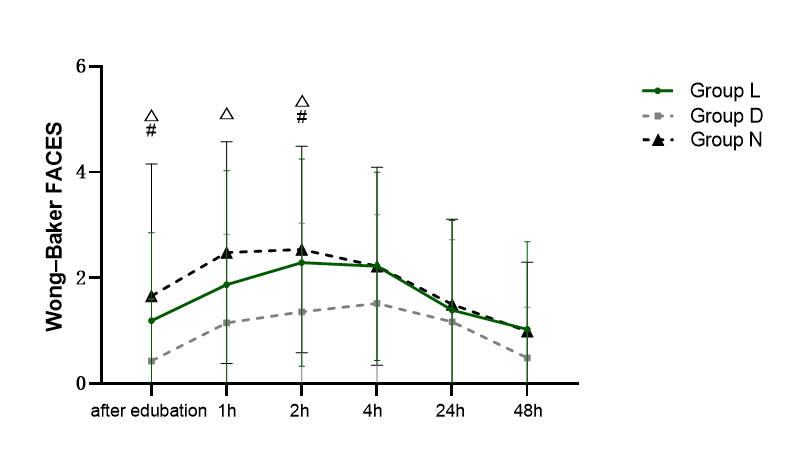


Supplementary Figure 4 Comparison of Wong Baker FACES pain scores of patients aged 1-12 years old at different time points (△p<0.05 comparison between group D and group N; #p comparison between group L and group D)


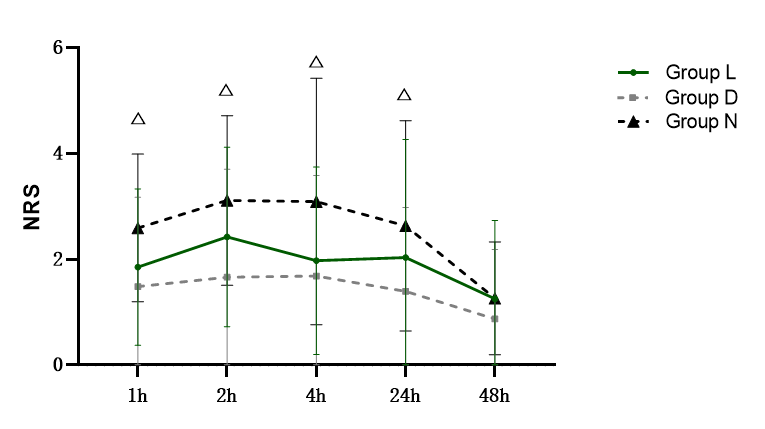


Supplementary Figure 5 Comparison of NRS pain scores of patients aged 7-12 years old at different time points (△p<0.05, comparison between group D and group N)
